# Cdc42 upregulation under high glucose induces podocyte apoptosis and impairs β-cell insulin secretion

**DOI:** 10.3389/fendo.2022.905703

**Published:** 2022-08-10

**Authors:** Shan Jiang, Chun-mei Xu, Shuai Yao, Rui Zhang, Xian-zhi Li, Ru-zhen Zhang, Tian-yue Xie, Yi-qian Xing, Qian Zhang, Xiao-jun Zhou, Lin Liao, Jian-jun Dong

**Affiliations:** ^1^ Department of Endocrinology, Qilu Hospital, Cheeloo College of Medicine, Shandong University, Jinan, China; ^2^ Department of Endocrinology, Shandong Provincial Hospital affiliated to Shandong University, Jinan, China; ^3^ Shandong Key Laboratory of Rheumatic Disease and Translational Medicine, Department of Endocrinology and Metabology, The First Affiliated Hospital of Shandong First Medical University & Shandong Provincial Qian Foshan Hospital, Shandong Institute of Nephrology, Jinan, China; ^4^ Department of Endocrinology and Metabology, Shandong Provincial Qianfoshan Hospital, Cheeloo College of Medicine, Shandong University, Jinan, China; ^5^ Department of Endocrinology, Shandong Provincial Qianfoshan Hospital, Shandong University of Traditional Chinese Medicine, Jinan, China; ^6^ Department of Pharmacology, Key Laboratory of Chemical Biology, School of Pharmaceutical Sciences, Shandong University, Jinan, China

**Keywords:** podocyte, Cdc42, apoptosis, co-culture, β-cells

## Abstract

**Objectives:**

The progressive impairment of β-cell function results in prolonged deterioration in patients with type 2 diabetes mellitus (T2DM). Interestingly, the finding on pancreatitis secondary to renal injury suggests that potential communication exists between kidney and pancreas. Therefore, we aimed to investigate cell division cycle 42 (Cdc42)-mediated podocyte apoptosis and its effect on insulin secretion in islet β-cells.

**Methods:**

Type 2 diabetic nephropathy mouse models were established to identify the expression of Cdc42 in podocytes by immunohistochemistry. An *in vitro* co-culture of mouse podocyte MPC5 and β-TC6 cells was preliminarily established. Subsequently, podocyte apoptosis induced by high glucose and Cdc42 was detected by TUNEL staining and western blotting. In addition, the JNK pathway was examined to determine the mechanism of apoptosis in MPC5 cells. Finally, insulin secretion and expression in β-TC6 cells as well as malondialdehyde (MDA) and superoxide dismutase (SOD) levels in both cell types were examined after the regulation of Cdc42 in MPC5 cells.

**Results:**

Cdc42 was highly expressed in the podocytes of diabetic nephropathy mice. Exposure to 25 mM glucose for 48 h induced a significant upregulation of Cdc42, Bax, and cleaved caspase-3 as well as a decreased Bcl-2 expression. In addition, marked apoptosis of MPC5 cells was observed compared to normal glucose treatment. After transfection with Cdc42 plasmid, apoptosis of MPC5 cells was enhanced with an increased expression of p-JNK, whereas inhibition of Cdc42 significantly alleviated podocyte apoptosis accompanied by a downregulation of p-JNK. The glucose-stimulated insulin secretion level of β-TC6 cells decreased after the upregulation of Cdc42 in MPC5 cells. Immunofluorescence staining for insulin showed that co-culture with MPC5 cells carrying the Cdc42 plasmid significantly reduced insulin expression, whereas inhibition of Cdc42 in MPC5 cells alleviated the above-mentioned abnormality of β-TC6 cells. The expression of Cdc42 and p-p38 in β-TC6 cells increased following the upregulation of Cdc42 in MPC5 cells; this was concurrent with augmented MDA levels and decreased SOD activity. The opposite result was observed for Cdc42 knockdown in MPC5 cells.

**Conclusions:**

Cdc42 in podocytes plays a crucial role in insulin secretion by β-cells, which may provide a new therapeutic target to prevent the vicious cycle of β-cell dysfunction in T2DM.

## Introduction

Type 2 diabetes mellitus (T2DM) is a metabolic disorder characterized by hyperglycemia due to insulin resistance, progressive islet β-cell dysfunction, and activation of an inflammatory cascade (1). The progressive impairment of β-cell function results in the prolonged and progressive deterioration of patients with the disease, and the current treatments for T2DM cannot satisfy the problematic situation in which β-cell function progressively worsens.

Existing studies have revealed the communication between various organs, such as heart and kidney in cardiorenal syndrome (2) and lung and kidney correlation (3). Traditional Chinese medicine (TCM), based on the viscus theory, considers the mutual breeding and transformation of the heart and kidney and their combined role in maintaining our lives and activities. In addition, studies have shown that some patients with massive polycystic kidney disease, chronic urinary tract infection, or refractory hypertension may develop traumatic pancreatitis during nephrectomy and kidney transplantation (4); however, the mechanism underlying pancreatitis after renal injury remains unclear.

Chuang et al. reported that kidney transplantation recipients were significantly susceptible to acute pancreatitis and poorer long-term outcomes, while alcohol consumption, gallstone disease, and a history of pancreatitis were significantly associated with acute pancreatitis risk after kidney transplantation (5). Furthermore, immunosuppressants in kidney transplant recipients may increase the risk of acute pancreatitis (6). Exploring the potential communication between the kidney and pancreas may be indeed a novel idea for the treatment of diabetes.

Podocytes are highly and terminally differentiated cells that play key roles in sustaining the integrity of the glomerular filtration barrier (7). The podocyte foot process disappears and fuses in the early stages of diabetic nephropathy (DN). Podocyte apoptosis is an early pathological mechanism leading to diabetic glomerular lesions and causes a decrease in podocyte number and density (8). Thus, finding an effective target to inhibit podocyte apoptosis is beneficial for alleviating DN injury.

Cell division cycle 42 (Cdc42) is a widely studied prototype of the Rho family and a powerful regulator of actin cytoskeletal dynamics, cell adhesion interactions, and motility (9). In addition, its other important functions include the regulation of gene expression, proliferation, and apoptosis (9, 10). Previous studies indicate that Cdc42 plays an important role in the pathogenesis of glomerular and other kidney diseases (11, 12). In addition, several studies have clarified the role of Cdc42 in the physiological function of the pancreas, particularly in mediating insulin secretion (13, 14); however, the role of Cdc42 in podocyte apoptosis, which promotes the functional deterioration of insulin secretion by β-cells, is not well understood. In the present study, we built a high glucose (HG)-induced co-culture model using a Transwell chamber to investigate Cdc42-mediated podocyte apoptosis and its influence on insulin secretion by islet β-cells; the effects of Cdc42 on HG-induced podocyte apoptosis were also evaluated. In addition, pancreatic β-cell function, including basal insulin secretion, glucose-stimulated insulin secretion, and insulin expression, was further explored by regulating Cdc42 expression in podocytes to reveal the potential role in pancreas–kidney communication, which is a vital and novel field for β-cell research.

## Materials and methods

### Construction of the animal model

Male C57BL/6J mice (6–8 weeks old) weighing 20 g were randomly divided into the sham operation group (Sham group), unilateral nephrectomy group (UNX group), and diabetic nephropathy group (DN group).

#### Preparation of unilateral nephrectomy mice model

The mice were restricted in diet but not restricted for water for 12 h before unilateral nephrectomy. After anesthesia, an ophthalmic scissor was used to make a transverse incision of about 1 cm in the back area. The incisions of the Sham group were sutured and closed immediately. After the tissue was incised in the UNX and DN groups, the right kidneys of the mice were fully exposed, and the adipose tissue and adrenal tissue around the kidney were bluntly separated with forceps. Subsequently, the right renal hilum vessels were ligated, and the right kidney was resected with ophthalmic scissors. The back muscles and fascia were then sutured.

#### Low-dose STZ intraperitoneal injection

The diabetes mellitus (DM) model was established by injecting streptozotocin (STZ, Solarbio, Beijing, China), which was dissolved in 0.1 M sodium citrate buffer (pH 4.5), to the mice in the DN group. The mice were injected intraperitoneally with STZ at a dose of 60 mg/kg daily for 5 consecutive days. After injection, the mice were given sufficient feed and purified water for 12 h, and then the mice were again restricted to diet but not to water for 12 h, after which they were injected with STZ again. The mice in the UNX group were also injected intraperitoneally but with sodium citrate buffer at the same dose. After 72 h of injection, the mice with random blood glucose higher than 16.7 mmol were considered successful diabetes models.

### Immunohistochemistry

Paraffin-embedded sections (4 μm) were deparaffinized with xylene and rehydrated using gradient concentrations of alcohol. The sections were placed in 0.01 M citrate buffer for high-pressure repair. Endogenous peroxidase activity was blocked using H_2_O_2_. Rabbit anti-Cdc42 primary antibodies (1:50; 10155-1-AP, Proteintech) were applied with overnight incubations at 4°C. After washing with phosphate-buffered saline (PBS), the sections were incubated with a goat anti-mouse HRP-conjugated secondary antibody (PV-9000, Zhongshan Golden Bridge) for 1 h. Finally, 3,3′-diaminobenzidine was used for color development.

### Cell lines and culture media

The clonal mouse cell line (β-TC6) and mouse podocyte cell line (MPC5) were purchased from Procell (Wuhan, China) and BeNa Culture Collection (Beijing, China), respectively. The MPC5 cells were propagated at 33°C and treated with 10 U/ml interferon-γ (IFN-γ) in Dulbecco’s modified Eagle’s medium (DMEM) (Invitrogen, USA), supplemented with 10% fetal bovine serum (FBS) (HyClone, Logan, UT), 100 units/ml penicillin, and 100 mg/L streptomycin. Subsequently, the cells were cultured at 37°C for 14 days without IFN-γ to induce differentiation. Synaptopodin, a podocyte differentiation marker, was used to identify MPC5 cell differentiation (**Supplementary Figure S1A**). Next, β-TC6 cells were maintained in DMEM containing 10% FBS, 100 units/ml penicillin, and 100 mg/ml streptomycin; they were incubated at 37°C in a 5% CO_2_ and 90% air humidified incubator.

### High glucose treatment

To determine the optimal HG concentration and treatment time, MPC5 cells were cultured for 24, 48, and 72 h in DMEM containing 5.5 mM glucose supplemented with 0, 10, 20, 30, 40, or 50 mM D-glucose (Sigma, Oakville, ON, Canada). MPC5 cells cultured in six-well plates with various glucose concentrations were collected to measure Cdc42 and apoptosis-associated proteins (Bax, cleaved caspase-3, and Bcl-2).

### Transfection of siRNAs and plasmids

siRNAs against Cdc42 and the negative control were designed and synthesized by GenePharma Co. Ltd. (Shanghai, China). Following the manufacturer’s protocol, they were transfected into MPC5 cells using Lipofectamine 2000 reagent (Invitrogen, Carlsbad, CA, USA). Opti-MEM (Gibco-BRL/Invitrogen, Carlsbad, CA, USA) containing 2.5 μg p-Cdc42 or an empty plasmid was mixed with 5 μl of Lipofectamine 3000 transfection reagent (Thermo Fisher Scientific, Waltham, MA, USA) and added to the cells. After 8–12 h, the Opti-MEM medium was removed, and the cells were again incubated with DMEM containing 10% FBS. Plasmid transfection-mediated Cdc42 upregulation was evaluated by RT-PCR and western blotting 48 h after transfection. Thus, MPC5 cells with stable upregulation and knockdown of Cdc42 (siCdc42) were generated. siNC was used as negative control.

The sense sequence for siCdc42 was 5′-GCAAGAGGAUUAUGACAGA-3′, while that of siNC was 5′- UUCUCCGAACGUGUCACGUTT-3′.

### TUNEL assay

Apoptotic cells were identified by the terminal deoxynucleotidyl transferase-mediated dUDP nick-end labeling (TUNEL) technique using an *in situ* cell death detection kit (Roche, Basel, Switzerland). Briefly, MPC5 cells were seeded on sterile glass coverslips in a six-well plate and stimulated with 5.5, 10, or 25 mM D-glucose for 48 h. The MPC5 cells were then washed with PBS and fixed with 4% paraformaldehyde. After permeabilization with 0.5% Triton X-100, the TUNEL assay was performed to detect the free 3-OH strand breaks resulting from DNA degradation. Additionally, MPC5 cells transfected with plasmids carrying *Cdc42*, siCdc42, and siNC were also stained by the TUNEL assay. The apoptosis rate in MPC5 cells was calculated by dividing the number of TUNEL-positive cells by the population of 100 counted cells per microscopic field (magnification: ×200) using an OLYMPUS FSX100 imaging system (Olympus, Tokyo, Japan).

### Co-culture model construction

When the density of β-TC6 cells reached 80–90%, the cells were digested, centrifuged, and resuspended in a full growth medium. The cells were seeded in six-well plates at a density of 5 × 10^4^ cells per well. Then, the MPC5 cells, including control MPC5 cells, MPC5 cells transfected with Cdc42-siRNA, negative control MPC5 cells transfected with siNC, and MPC5 cells transfected with the Cdc42 plasmid, were treated as detailed above, except for seeding in the Transwell chamber. The chamber was then removed from the six-well plates with tweezers. Transwell cell culture inserts (catalog number: 3470; pore size: 0.4 μm; Corning Costar Corp., NY, USA) were placed in DMEM with 10% FBS and 1% antibiotics (100 units/ml penicillin and 100 mg/ml streptomycin) in both the upper and lower compartments. The media in the upper and lower compartments were maintained at similar levels to avoid bulk flow caused by a hydrostatic pressure gradient. The entire system allowed mutual communication between different cells through a polyester membrane for 48 h. As a control, β-TC6 cells were monocultured in six-well plates without inserts.

### Insulin release studies

After the co-culture with MPC5 cells for 48 h and at 80–90% confluence, the β-TC6 cells were washed with glucose-free Krebs/HEPES Ringer (KRBH) solution twice and preincubated at 37°C for 30 min with the glucose-free KRBH solution. The β-TC6 cells were then incubated with basal KRBH solution containing 3.3 mM glucose for 1 h, and the supernatants were collected for baseline insulin release. Subsequently, the β-TC6 cells were incubated with HG (16.7 mM) KRBH solution for 1 h, and the supernatants were collected to measure the stimulated insulin release.

Insulin released into the medium was quantified by ELISA and expressed in nanogram per milliliter. Briefly, standard insulin solutions of 0 ng/ml (5 ml) and 1, 2.5, 5, 10, 20, and 50 ng/ml (0.1 ml) were prepared. Then, 5 µl of the standard or sample was added to the well, after which 100 µl of 1× detection antibody solution was added to each well. The plate was sealed with a plate cover, incubated at room temperature for 90 min, and shaken at 600 rpm. The contents were discarded, and the plate was tapped on a clean paper towel to remove the residual solution from each well. Next, 300 µl of 1× wash buffer was added to each well and incubated at room temperature for 20 s. After discarding the wash buffer, the plate was tapped on a clean paper towel to remove the residual wash buffer; this washing step was repeated four times. The substrate solution (100 µl) was added to each well and incubated at room temperature for 15 min. Finally, 100 µl of stop solution was added to each well, and the plate frame was gently tapped for a few seconds to ensure thorough mixing. The absorbance of each well was then measured at 450 nm.

### Immunofluorescence staining for insulin

For immunofluorescence, β-TC6 cells were seeded on sterile glass coverslips in the lower chamber of the Transwell system or a single six-well plate and co-cultured with different MPC5 cells. Glass coverslips containing β-TC6 cells were then extracted to detect the immunofluorescence intensity. Slides were fixed for 30 min with 4% paraformaldehyde, followed by permeabilization with 0.5% Triton X-100 in PBS for 5 min. The slides were then incubated with the insulin antibody (1:100; 15848-1-AP, Proteintech) at 4°C overnight. On the following day, Alexa Fluor 594-conjugated secondary antibodies (1:50; Invitrogen, CA, USA), along with DAPI staining to visualize the nuclei, were used to detect the immunoreactive products of insulin. After staining, images were taken at randomly selected fields using an OLYMPUS FSX100 imaging system.

### Measurements of superoxide dismutase

Superoxide dismutase (SOD) activity was measured using an SOD assay kit (Beyotime, Shanghai, China). The cells were washed with precooled PBS and resuspended in 150 µl of sample preparation solution per one million cells. Briefly, 20 µl of the sample was added to 96-well culture plates, and the SOD assay buffer was used as a blank control. Then, the WST-8/enzyme working liquid and reaction start-up reagent were added. After 30 min of incubation at 37°C, absorbance was detected at 450 nm.

### Measurements of malondialdehyde

The MDA content was measured using an MDA assay kit (Beyotime, Shanghai, China). After centrifugation at 12,000 × *g* for 10 min at 4°C, the supernatants were collected. A total of 200 µl of the MDA working solution was added to the samples and standards. After heating at 100°C for 15 min, the mixture was cooled to room temperature and centrifuged for 10 min at 1,000 × *g*. Then, 200 µl of the mixture was added to a 96-well plate, and the absorbance was measured at 532 nm using a microplate reader.

### Western blotting

Cells were lysed with RIPA lysis buffer (Beyotime Biotechnology, Shanghai, China) for 30 min on ice and then centrifuged at 12,000 × *g* for 30 min at 4°C. The supernatant was extracted, and the protein concentration was measured using a bicinchoninic acid (BCA) assay kit (Thermo Fisher Scientific, Waltham, MA, USA). Briefly, standards with concentrations of 0, 0.025, 0.05, 0.1, 0.2, 0.3, 0.4, and 0.5 mg/ml as well as 20 µl of the protein sample were prepared and added to the wells. Next, 200 µl of BCA working reagent was added to each well, and the mixtures were incubated at 37°C for 30 min. The absorbance of the samples was measured at 562 nm using a microplate reader.

The protein extracts were boiled with SDS loading buffer (Beyotime, Shanghai, China) and separated using SDS-PAGE. Western blotting was performed with the antibodies against Cdc42 (1:1,000; 10155-1-AP, Proteintech), synaptopodin (1:1,000; 21064-1-AP, Proteintech), Bax (1:1,000; GB11007, Servicebio), cleaved caspase-3 (1:1,000; 9664S, Cell Signaling Technology), Bcl-2 (1:1,000; 26593-1-AP, Proteintech), JNK (1:1,000; 9252, Cell Signaling Technology), phospho-JNK (1:1,000; AF3320, Affinity), p38 (1:1,000; ET1702-65, HUABIO), and phospho-p38 (1:1,000; AF4001, Affinity). Antibodies against Cdc42 (1:1,000; 10155-1-AP, Proteintech) were used to examine transfection efficiency with the siCdc42 and Cdc42 plasmids. β-actin (1:5,000; 60008-1-Ig, Proteintech) was used as the reference protein.

### Quantitative real-time PCR

Total RNA was extracted from the cultured cells using TaKaRa RNAiso Plus (catalog number: 9108) according to the manufacturer’s protocol. A microgram of RNA from each sample was added to a 20-μl reaction mixture, and cDNA was synthesised using the PrimeScript™ RT reagent Kit with a genomic DNA Eraser (catalog number: RR047A, Takara). Quantitative real-time PCR was performed using UltraSYBR Mixture (low ROX) (CWBIO, Inc., Beijing, China) to detect Cdc42 and Bax mRNA expression.

β-actin was used as an internal control. The 2^-△△CT^ method was used to calculate the relative expression level of each mRNA. The expression levels were normalized against those of β-actin. The primer sequences are shown in **Table 1**.

**Table 1:** Primer sequences.

Gene	Forward (5′–3′)	Reverse (5′–3′)
Cdc42	ATTATGACAGACTACGACCGCT	AGTGGTGAGTTATCTCAGGCA
Bax	GATGGCAACTTCAACTGGGG	CAGCCACCCTGGTCTTGG
β-actin	GGCTGTATTCCCCTCCATCG	CCAGTTGGTAACAATGCCATGT

### Statistical analysis

All statistical analyses were performed using SPSS Statistics 22.0 (SPSS Inc., Chicago, IL, USA). Student’s *t*-test was used to assess the significance of data within the two groups. Multiple statistical comparisons were performed using one-way ANOVA variance followed by *post-hoc* tests. Data are presented as means ± standard error of the mean (SEM), and the level of statistical significance was estimated at *P <*0.05.

## Results

### Cdc42 was highly expressed in the podocytes of mice with DN

The expression of Cdc42 was examined, and the results showed that Cdc42 was upregulated in the kidneys of the DN group when compared with the UNX and Sham groups (**Supplementary Figure S1B**). In addition, immunohistochemical analysis suggested that Cdc42 was increased in the podocytes of mice in the DN group compared to the UNX and Sham groups (**Supplementary Figure S1C**).

### HG increased the Cdc42 levels, accompanied by the upregulation of Bax and cleavage of caspase-3 as well as the downregulation of Bcl-2 in MPC5 cells

**Figure 1:**
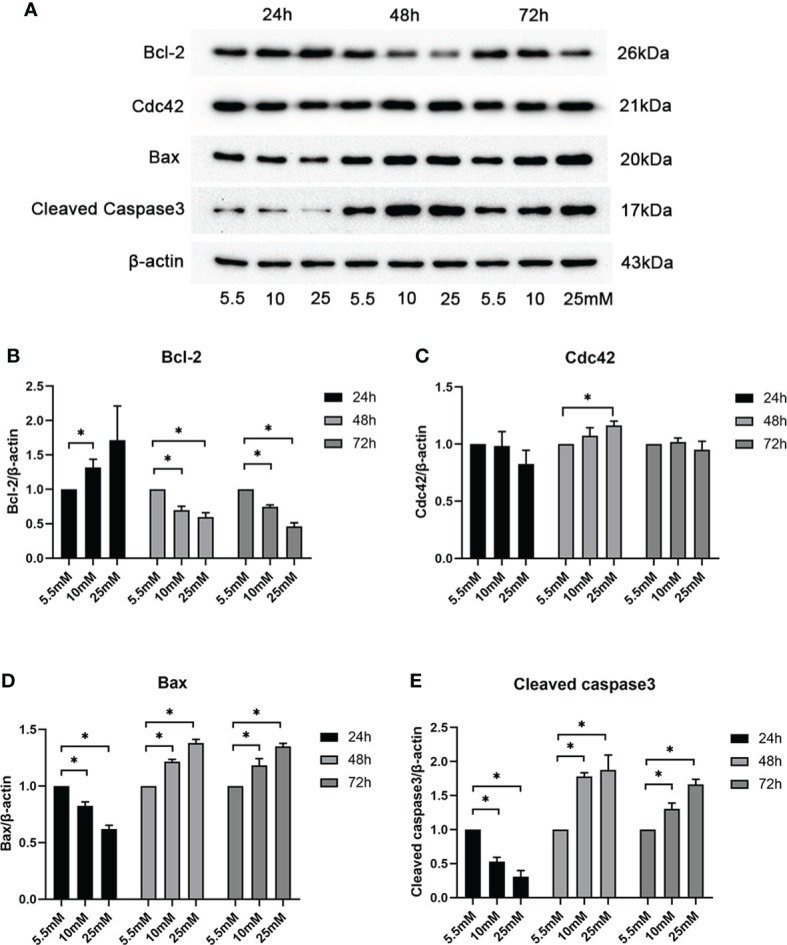
Examination of Cdc42 and Bax when treated with various glucose concentrations at different timepoints. (**A–E**) The protein expression levels of Cdc42, Bax, and cleaved caspase-3 increased, while those of Bcl-2 decreased, when treated with 25 mM glucose for 48 h. Data are presented as mean ± SEM; *P < 0.05.

To determine whether and how high glucose levels affect podocyte apoptosis, we cultured mouse MPC5 cells in DMEM with normal (5.5 mM) and various high concentrations (10, 25, 30, 40, and 50 mM) of glucose for 24, 48, and 72 h. The specific effect of HG on apoptosis was determined by the Bax protein levels, a well-recognized indicator of apoptosis. As shown by western blotting analysis (**Supplementary Figure S2**), HG (25 mM) significantly increased the abundance of Bax compared with the control, suggesting that 25 mM was the optimal concentration of glucose to induce apoptosis in MPC5 cells. A significant increase in Cdc42 was observed when the cells were treated with 25 mM HG compared to the 5.5-mM-glucose group. To confirm the optimal intervention time, we divided the groups into 5.5, 10, and 25 mM HG for 24, 48, and 72 h. In line with the results mentioned above, the western blotting analysis showed that treatment with 25 mM HG for 48 h dramatically upregulated the Cdc42 levels in MPC5 cells, accompanied by the overexpression of Bax and cleaved caspase-3 as well as the downregulation of Bcl-2 (**Figures 1A–E**).

### MPC5 cell apoptosis significantly increased in 25 mM HG

**Figure 2:**
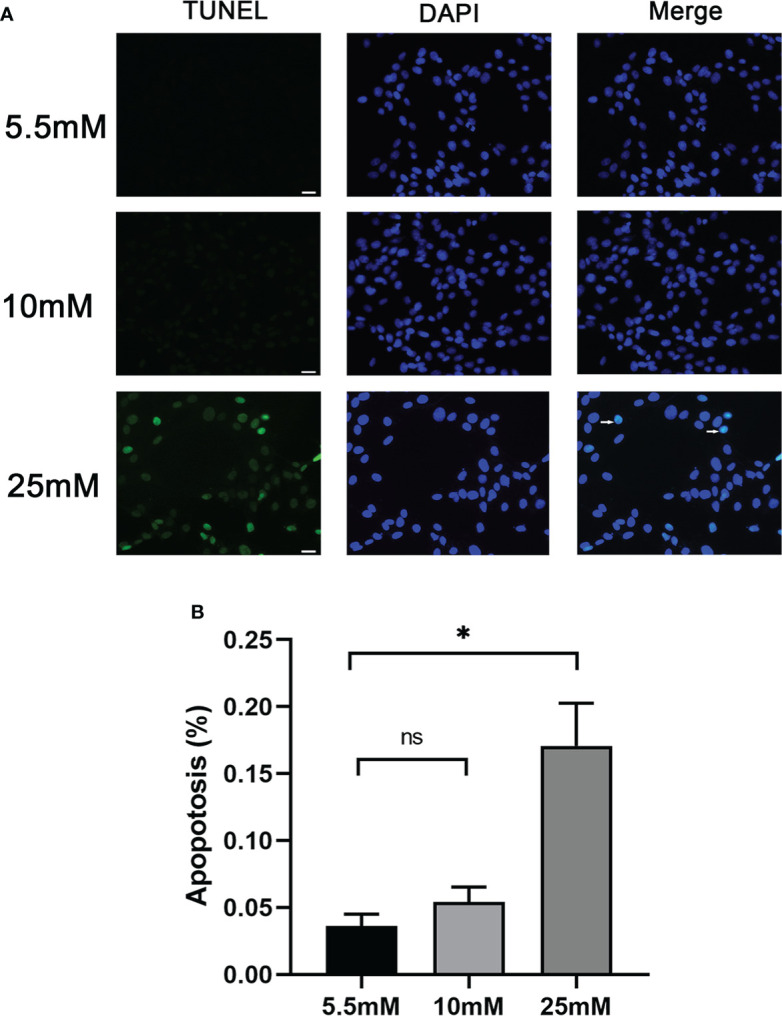
Podocyte apoptosis was detected by TUNEL staining with different glucose concentrations. **(A)** Podocyte apoptosis rate increased significantly in response to high glucose (25 mM). White arrows point to TUNEL-positive podocytes (apoptotic podocytes); magnification: ×200; scale bar = 25 μm. **(B)** Absolute count of TUNEL-positive podocytes. Data were expressed as the mean numbers of apoptotic podocytes from 20 randomly selected fields and were from at least three independent experiments. Data are presented as mean ± SEM; *P < 0.05; ns, not significant.

Cell apoptosis was further evaluated by *in vitro*-cultured MPC5 cells treated with 10 and 25 mM HG. The measurement of apoptotic cells by TUNEL staining (**Figure 2**) showed that the number of MPC5 apoptotic cells was increased when treated with 25 mM HG compared to the control group (0.17 ± 0.55 *versus* 0.04 ± 0.02, *P* < 0.05, **Figure 2**). However, no difference was observed between the 10-mM-glucose group and the control group (0.05 ± 0.02 *versus* 0.04 ± 0.02, **Figure 2**).

### Cdc42 upregulation augmented Bax protein in MPC5 cells

**Figure 3:**
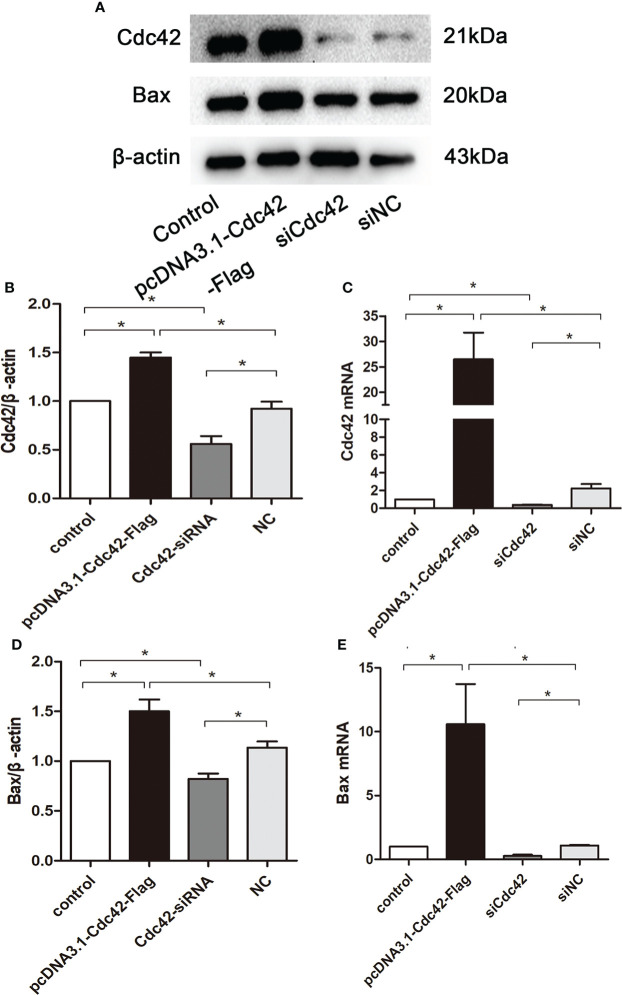
Upregulation of Cdc42 increased the Bax levels in MPC5 cells. (**A**, **B**, **D**) The protein expression levels of Cdc42 and Bax were detected. When transfected with a plasmid carrying the *Cdc42* gene, the Cdc42 and Bax proteins were significantly increased compared to the negative control, whereas transfection with Cdc42-siRNA reduced the Cdc42 and Bax protein levels. (**C**, **E**) The mRNA expression of Cdc42 and Bax increased in plasmid-transfected podocytes compared to the control; conversely, the mRNA expression of Cdc42 and Bax decreased in siCdc42-transfected podocytes. The data were from at least three independent experiments and are presented as mean ± SEM; *P < 0.05.

To further explore the role of increased Cdc42 levels in podocyte apoptosis, we studied the effect of Cdc42 on MPC5 apoptosis *in vitro*. As shown in **Figures 3A–C** the Cdc42 protein and mRNA expression were markedly increased when transfected with Cdc42 plasmid compared to the negative control; however, these levels decreased upon transfection with siCdc42 compared to siNC in MPC5 cells. We also found that the Bax mRNA and protein expression levels were significantly increased in Cdc42-upregulated MPC5 cells, whereas a noticeable decline in Bax levels was found when treated with siCdc42 in MPC5 cells (**Figures 3D, E**).

### Loss of Cdc42 significantly alleviated the HG-induced podocyte apoptosis with a decreased p-JNK/JNK ratio

**Figure 4:**
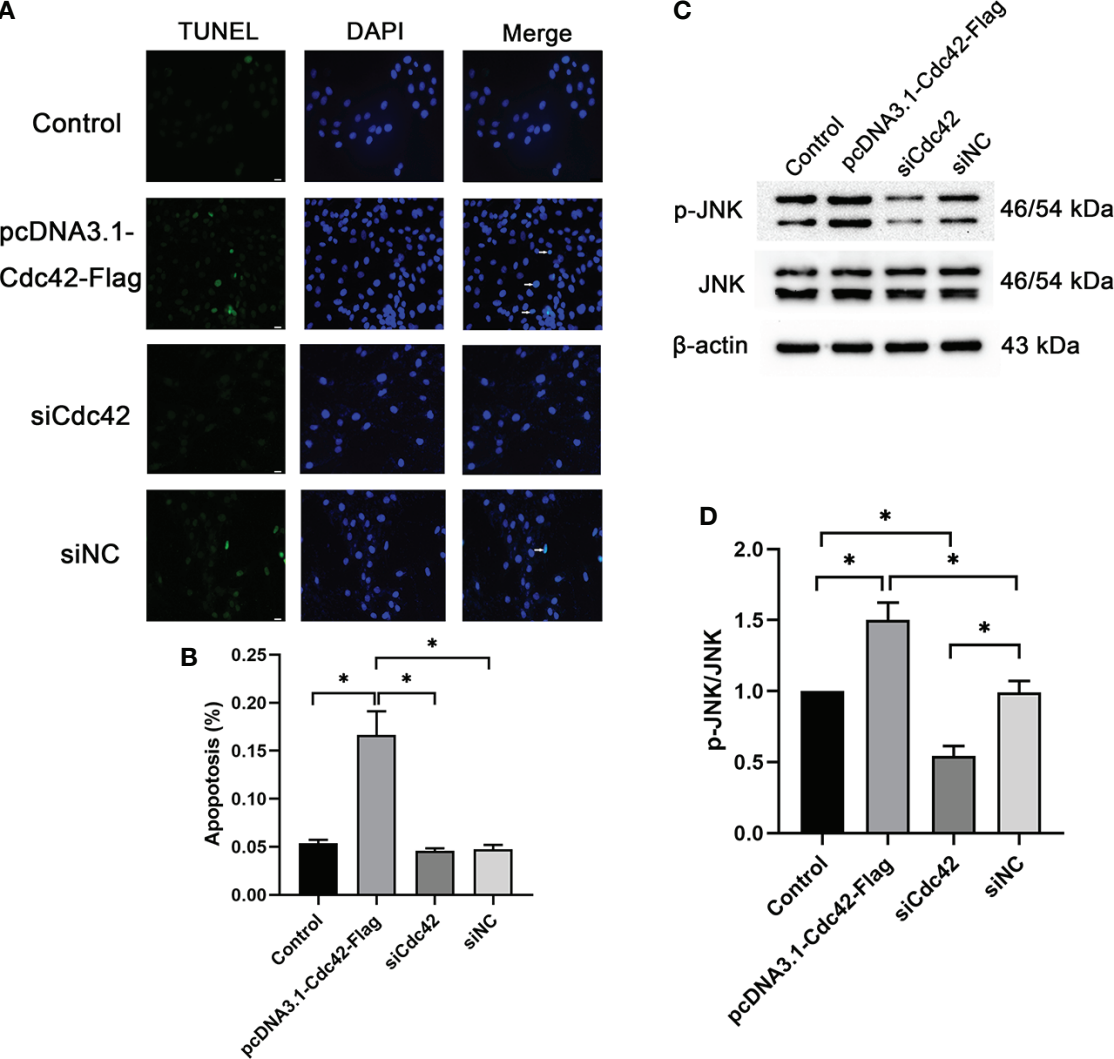
Upregulation of Cdc42-induced podocyte apoptosis. **(A)** The control podocytes, podocytes transfected with the plasmid carrying the *Cdc42* gene, podocytes transfected with Cdc42*-*siRNA, and podocytes transfected with negative control siRNA were cultured in a medium containing glucose (5.5 mM). White arrows point to TUNEL-positive podocytes (apoptotic podocytes); magnification: ×200; scale bar = 25 μm. **(B)** The apoptosis rate (%) was calculated by dividing the number of TUNEL-positive cells by a population of 100 counted cells per condition. The podocyte apoptosis rate increased significantly after Cdc42 upregulation. The data were from at least three independent experiments. **(C, D)** The protein expression levels of JNK and p-JNK were detected. When transfected with the plasmid carrying the *Cdc42* gene, the p-JNK/JNK ratio decreased significantly compared to the negative control, whereas transfection with Cdc42-siRNA increased the p-JNK/JNK ratio. The data are means ± SEM; *P < 0.05.

We further surveyed podocyte apoptosis by the TUNEL assay after regulating Cdc42. As shown in **Figures 4A, B** and consistent with the results of the Bax expression mentioned above, the cell apoptosis rate was markedly increased in the *in vitro*-cultured MPC5 cells transfected with Cdc42 plasmid compared with the negative control group (0.17 ± 0.43 *versus* 0.05 ± 0.08, *P* < 0.05). However, after the loss of Cdc42, the cell apoptosis rate was significantly alleviated in MPC5 cells (0.05 ± 0.05 *versus* 0.17 ± 0.43, *P* < 0.05, **Figure 4**). These results indicated that Cdc42 mediates podocyte apoptosis under HG conditions.

It is well established that the JNK pathway plays an essential role in apoptosis (15, 16). Therefore, to investigate whether Cdc42 contributes to podocyte apoptosis through the JNK pathway, JNK and p-JNK were measured following the regulation of Cdc42 for 24, 48, and 72 h. The effect of Cdc42 on the JNK pathway is not obvious at 24 h (**Supplementary Figures S3A, B**). After 72 h of transfection, p-JNK was increased in MPC5 cells transfected with Cdc42 plasmid compared with the negative control group, while no statistically significant reduction in p-JNK expression was found when downregulating Cdc42 (**Supplementary Figures S3E, F**). The results showed that, at 48 h after transfection, Cdc42 overexpression facilitated the expression of p-JNK. Conversely, the Cdc42 knockdown resulted in opposite effects (**Figures 4C, D**), which supported the involvement of the JNK pathway in Cdc42-mediated podocyte apoptosis.

### Reduced insulin expression and secretion and increased Cdc42 expression and p-p38/p38 ratio were observed in β-TC6 cells when co-cultured with MPC5 cells after Cdc42 regulation

**Figure 5:**
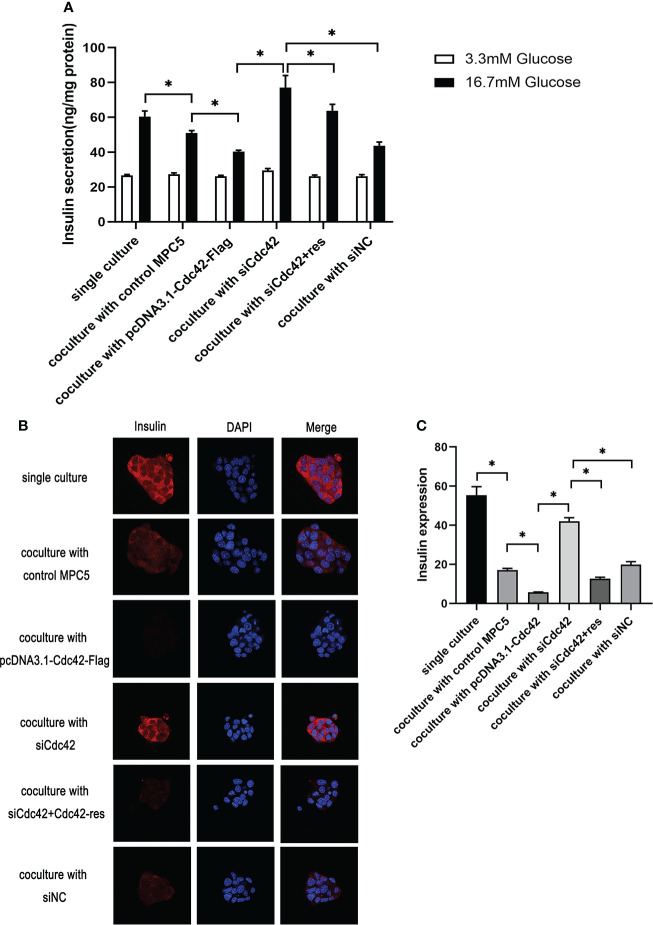
Upregulation of Cdc42 in podocytes impaired glucose-stimulated insulin secretion (GSIS) and insulin expression in β-TC6 cells. **(A)** A co-culture with MPC5 cells resulted in impaired insulin secretion from β-TC6 cells stimulated with 16.7 mM glucose compared with the monocultured group. The upregulation of Cdc42 in MPC5 cells diminished the GSIS of β-TC6 cells compared to the siNC group without a change in basal insulin secretion, while the impaired GSIS of β-TC6 cells was reversed by co-culture with MPC5 cells transfected with siCdc42. However, insulin secretion was partially restored when cotransfected with siCdc42 and Cdc42-res in MPC5 cells. **(B)** Immunofluorescence staining for insulin (red dots) showed a lower expression in the β-TC6 cells when co-cultured with MPC5 cells compared with monocultured β-TC6 cells. The upregulation of Cdc42in MPC5 cells significantly reduced the insulin expression in β-TC6 cells compared to the siNC group. When Cdc42 was suppressed in MPC5 cells, the insulin level was significantly upregulated in β-TC6 cells. Nevertheless, this effect was counteracted when co-cultured with MPC5 cells transfected with siCdc42 and Cdc42-res; original magnification: ×400. **(C)** The quantitative expression of insulin was examined, and no significant difference was observed. The data are presented as mean ± SEM; *P < 0.05.

We measured the glucose-stimulated insulin secretion (GSIS) and insulin expression to evaluate the role of Cdc42 in MPC5 cells on pancreatic β-cell dysfunction. To better understand Cdc42, an siRNA-resistant Cdc42 vector was constructed to express Cdc42 protein (Cdc42-res). After the transfection of MPC5 cells with siCdc42, Cdc42 plasmid, siCdc42+Cdc42-res, or siNC, the β-TC6 cells were co-cultured with various MPC5 cells and then stimulated with 3.3 or 16.7 mM glucose, after which the amount of secreted insulin was measured as the basal insulin secretion (BIS) or GSIS, respectively. As shown in **Figure 5**, co-culture with MPC5 cells impaired insulin secretion by β-TC6 cells stimulated with 16.7 mM glucose compared with the monoculture of β-TC6 cells (*P* < 0.05). Additionally, the upregulation of Cdc42 in MPC5 cells diminished the insulin secretion from β-TC6 cells stimulated with 16.7 mM glucose compared to the siNC group (*P* < 0.05), without any change in BIS. This damaging effect of Cdc42 on the GSIS of β-TC6 cells was reversed by coculturing with MPC5 cells transfected with siCdc42 (*P* < 0.05). However, insulin secretion was partially restored when the MPC5 cells were co-transfected with siCdc42 and Cdc42-res. Additionally, normal insulin expression, indicated by red dots, was observed in monocultured β-TC6 cells (**Figure 5**). The co-culture with control MPC5 cells for 48 h significantly reduced the insulin level in β-TC6 cells compared with that in the monocultured group (**Figures 5B, C**). When co-cultured with MPC5 cells transfected with an overexpressed Cdc42 plasmid, we found a decreased insulin expression in the β-TC6 cells; however, a significant improvement in insulin levels was achieved by inhibiting Cdc42 expression in MPC5 cells. Nevertheless, this effect was counteracted when co-cultured with MPC5 cells co-transfected with siCdc42 and Cdc42-res (**Figures 5B, C**).

**Figure 6:**
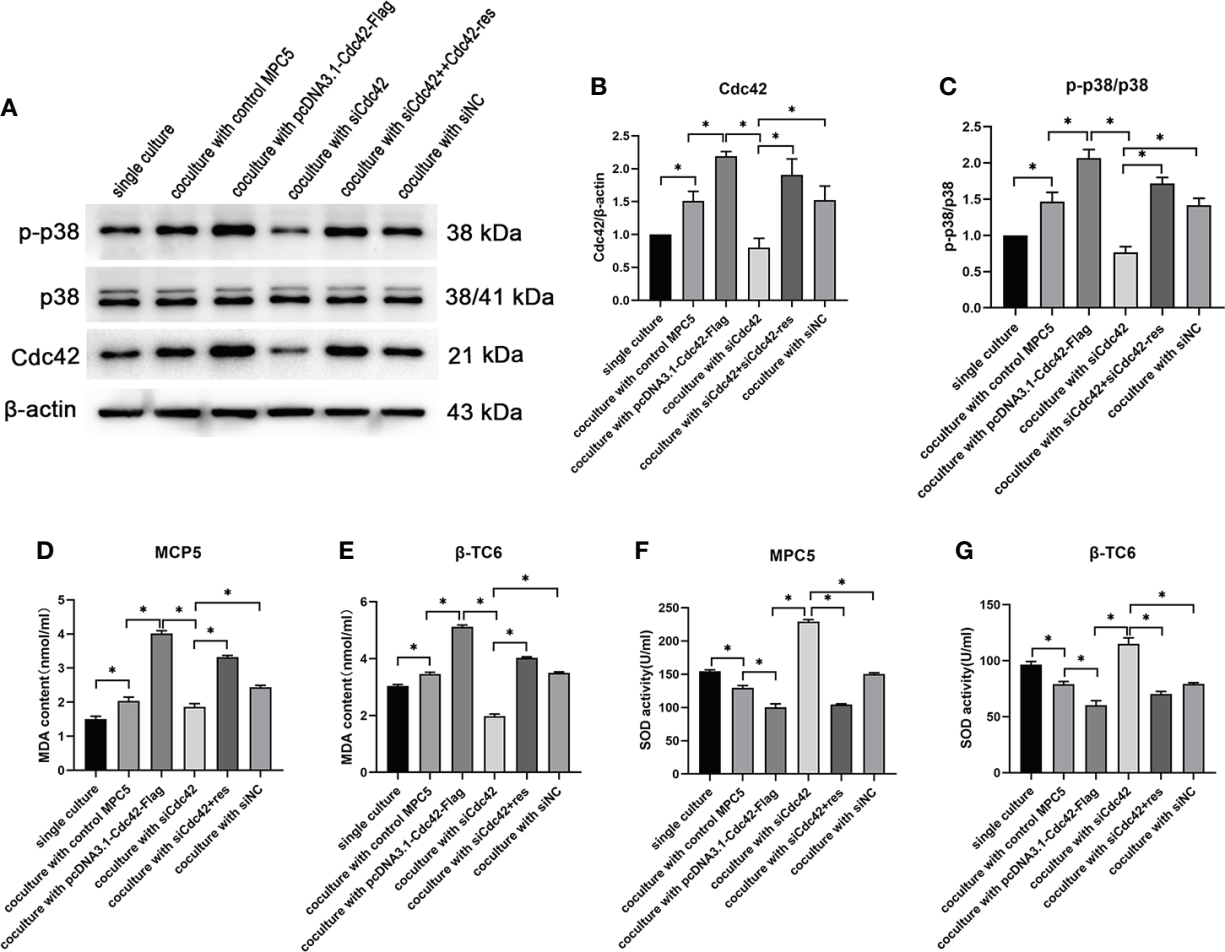
The upregulation of Cdc42 in podocytes increased the Cdc42 expression and the p-p38/p38 ratio in β-TC6 cells, which were accompanied by an increase in malondialdehyde (MDA) levels and a decrease in SOD activity. (**A–C**) The protein expression levels of Cdc42, p-38, and p-p38 in β-TC6 cells were detected. (**D–G**) The levels of MDA and SOD in MPC5 cells and β-TC6 cells were measured. Data are presented as mean ± SEM; *P < 0.05.

Given that p38 can act as a receptor of Cdc42, the expression of Cdc42 and the p-p38/p38 ratio were examined in β-TC6 cells after co-culture with MPC5 cells. β-TC6 cells co-cultured with control MPC5 cells showed an increased expression of Cdc42 and p-p38 compared to the monocultured group. When co-cultured with Cdc42-overexpressing MPC5 cells, the expression levels of Cdc42 and p-p38 were upregulated in β-TC6 cells. The opposite results were obtained when Cdc42 was knocked down in MPC5 cells, which could be reversed by the addition of Cdc42-res (**Figures 6A–C**).

### Levels of oxidative stress were enhanced in both β-TC6 and MPC5 cells after co-culture concomitantly with Cdc42 regulation

To elucidate whether oxidative stress functions in the co-culture process regulating Cdc42, the levels of MDA and SOD were measured in MPC5 and β-TC6 cells, respectively. We observed increased MDA levels and decreased SOD activity compared to β-TC6 cell monoculture when co-cultured with control MPC5 cells. Upon co-culture with MPC5 cells transfected with the overexpressed Cdc42 plasmid, the level of MDA was further augmented, while SOD activity was further reduced. In contrast, when co-cultured with Cdc42 knockdown cells, we observed reduced levels of MDA and enhanced SOD activity, which were reversed by additional transfection with Cdc42-res (**Figures 6D–G**).

## Discussion

Type 2 diabetes, the most common type of DM, highly correlates with progressive β-cell failure, which manifests as combined insulin secretory dysfunction and insulin resistance (17). However, the molecular mechanisms underlying the coupling of glucose-stimulated insulin secretion are only partially understood. In the present study, we examined whether Cdc42, a key factor that mediates podocyte apoptosis, can induce insulin dysfunction in pancreatic β-cells. The inhibition of Cdc42 in MPC5 cells markedly alleviated impaired GSIS and decreased insulin expression induced by increased Cdc42 in podocytes stimulated by HG, suggesting an indirect negative modulatory role for Cdc42 on insulin secretion from pancreatic β-cells. These results demonstrate the significance of Cdc42 regulation in podocytes for β-cell secretory function from the unique perspective of communication with different cells.

Due to apoptosis, podocyte loss, which has been reported in patients with DM with or without DN, is a crucial component and the strongest indicator of progressive kidney injury (18, 19). In our previous research, a significant cytoskeletal reorganization of podocytes was observed under HG conditions accompanied by loss of cytoskeleton-related protein synaptopodin, but the mechanism involved remains to be explored.

It is well established that Cdc42, a small GTPase of the Rho family, plays a vital role in podocyte cytoskeletal remodeling (20); however, the role of Cdc42 in the progression of DN is still controversial. Cdc42 is believed to contribute to DN, especially in podocyte injuries, due to its impaired function in the podocyte cytoskeleton (14). Recently, it has been shown that indoxyl sulfate exposure enhanced the phosphorylation of Cdc42, increasing the cytoskeletal dynamics in mouse podocytes (21). A previous study has also shown cytoskeletal rearrangement and loss of arch F-actin fibers in podocytes—after exposure to angiotensin II (Ang II) or TGF-β1—by enhancing the activity of Cdc42 (22). On the contrary, other studies have suggested the role of SRGAP2a in stabilizing the podocyte cytoskeleton *via* its interaction with Cdc42 (23). Therefore, the potential regulatory role of Cdc42 in HG-induced podocyte injury was investigated in this study. We found that apoptosis was significantly induced by increased Cdc42 levels as detected by TUNEL staining. We also found that upregulation of Cdc42 by HG promoted the increase in Bax and cleaved caspase-3 levels, which are apoptosis-related proteins in podocytes, but the suppression of Cdc42 blocked the effect. This further suggests that upregulation of Cdc42 is implicated in HG-induced podocyte apoptosis. The abovementioned findings were inconsistent with the research conducted by Huang et al. (24), which may be due to the different experimental techniques, cell line origin, experimental design, or HG concentration. It is plausible to speculate that the upregulation of Cdc42 may result in decreased stabilization and rearrangement of the cytoskeleton and even contribute to the decline in adhesion and apoptosis of podocytes (14).

JNK is a member of the mitogen-activated protein kinase superfamily, which can be activated by phosphorylation of the N-terminal Ser-63 and Ser-73 residues (25, 26). The JNK pathway is an important regulatory pathway in apoptosis (27). Our results showed that Cdc42 induced the apoptosis of MPC5 cells partly through the JNK pathway, which is consistent with a previous study that demonstrated that gasdermin D knockdown attenuated HG-induced apoptosis by inhibiting the phosphorylation of JNK (26).

Typically, pancreatic β-cells release sufficient insulin to maintain glucose homeostasis in response to elevated blood glucose levels. Dysfunction of the secretory response is considered a causal factor in the etiology of T2DM. Diabetes affects various tissues and organs, including the kidney, skeletal muscle, eyes, islets, and brain (28–34). Moreover, existing evidence suggests that the transport of vehicles containing secretory insulin granules is involved in a well-coordinated crosstalk between various signaling proteins and molecules through changes in the cytoskeletal architecture—emphasising the critical role of communication between cells and molecules in the pathogenesis of diabetes. Furthermore, simultaneous pancreas/kidney (SPK) transplants, the most common pancreas transplant category, have been generally accepted by diabetic patients with uremia. Existing evidence has revealed that SPK transplantation has been shown to preserve long-term kidney graft function better than the transplantation of a kidney alone (35) and is not only life-enhancing but also life-saving (36), which indicates a potential correlation between the pancreas and kidney. However, the mechanism involved in the beneficial effects of SPK transplantation on patient survival and quality of life remains unclear.

Combined with the current situation of SPK research, we designed a co-culture model to explore the communication between podocytes and β-cells and further investigate the effects of Cdc42 changes in podocytes on the insulin secretory function of β-cells. Interestingly, the GSIS was markedly decreased by the upregulation of Cdc42 in podocytes without a change in BIS *in vitro*, which suggests a potential influence of Cdc42 in podocytes on GSIS of β-cells.

Subsequently, our results illustrated that the upregulation of Cdc42 in MPC5 cells induced the corresponding expression of Cdc42, and its receptor p-p38/p38 was elevated in β-TC6 cells accompanied by reduced insulin secretion. Previous studies showed that Cdc42 plays an essential role in the second/sustained phase of insulin secretion by regulating PAK-1 (14, 37) and participates in the targeting of insulin-containing granules to syntaxin 1A, one of the SNARE proteins, while it also contributes to the specific targeting of insulin granules to sites of exocytosis (38), which is not in agreement with our results; a potential reason may be the involvement of other pathways. Yang et al. demonstrated that methylmercury treatment induced oxidative stress-mediated pancreatic β-cell apoptosis (15). In our study, the levels of oxidative stress in both cell types were evaluated, and the results showed that the upregulation of Cdc42 in MPC5 cells enhanced oxidative stress in MPC5 cells and β-TC6 cells. During co-culture between MPC5 cells and β-TC6 cells, not only Cdc42 but also other cell-to-cell communications (such as those *via* exosomes) may contribute to insulin secretion. The indirect effect of Cdc42 changes in other cells on insulin secretion has not yet been elucidated. Based on previous findings and our present results, we speculated that Cdc42, a master regulator of cytoskeletal dynamics and cell polarity in mouse podocytes, plays a critical regulatory role in insulin secretion; we also revealed an intimate association between renal damage and insulin secretion.

This study has some limitations that must be addressed. First, it was not determined whether MPC5 cells affected β-TC6 cells through cell-to-cell communication. In addition, we did not perform an experiment to modulate the JNK pathway. Further investigations are needed to gain insight into the underlying mechanisms.

## Conclusions

We showed that Cdc42 functions as a regulator of diabetes-associated tissue injury, manifesting as an insulin secretion disorder with podocyte apoptosis. Although Cdc42 is highly discussed, the exact connection between renal podocytes and β-cells remains elusive, and further experiments are required. Nevertheless, modulation of Cdc42 activity and its regulatory pathways might serve as a therapeutic target to prevent a vicious cycle in which kidney damage and impaired insulin secretion interact with each other and ultimately lead to an aggravated outcome.

## Data availability statement

The original contributions presented in the study are included in the article/**Supplementary Material**. Further inquiries can be directed to the corresponding authors.

## Ethics statement

The animal study was reviewed and approved by the Ethics Committee on Science Research of Shandong University Qilu Hospital.

## Author contributions

The study was conceived and designed by LL and J-JD. SJ performed experiments and interpreted the results. C-MX, SY, T-YX, R-ZZ, and Y-QX assisted in conducting the experiments and analyzed the data. SJ and X-JZ drafted the manuscript. QZ, X-ZL, and RZ edited the figures. All authors contributed to the article and approved the submitted version.

## Funding

This work was supported by grants from the National Natural Science Foundation of China (grant numbers: 82170847, 81770822, and 82170824).

## Conflict of interest

The authors declare that the research was conducted in the absence of any commercial or financial relationships that could be construed as a potential conflict of interest.

## Publisher’s note

All claims expressed in this article are solely those of the authors and do not necessarily represent those of their affiliated organizations, or those of the publisher, the editors and the reviewers. Any product that may be evaluated in this article, or claim that may be made by its manufacturer, is not guaranteed or endorsed by the publisher.

## Supplementary material

Supplementary Figure 1 Identification of MPC5 cell differentiation and the expression of Cdc42 in mice with or without diabetic nephropathy (DN). (**A**) The protein expression of synaptopodin. (**B**) The protein expression of Cdc42 in the UNX, Sham, and DN groups. (**C**) Immunohistochemical evaluation of Cdc42 in the UNX, Sham, and DN groups; original magnification: ×400.

Supplementary Figure 2 The expression of Cdc42 and Bax at various glucose concentrations and timepoints. Cdc42 and Bax protein levels when treated with various glucose concentrations at different timepoints. High glucose (25 mM) significantly increased the abundance of Cdc42 and Bax compared with normal glucose.

Supplementary Figure 3 Protein expression levels of JNK and p-JNK at different time gradients. The protein expression levels of JNK and p-JNK were detected at 24, 48, and 72 h after transfection. UNX, unilateral nephrectomy group; Sham, sham operation group; DN, diabetic nephropathy. The data are presented as mean ± SEM; **P* < 0.05.

## Abbreviations

T2DM, type 2 diabetes mellitus; GSIS, glucose-stimulated insulin secretion; TCM, traditional Chinese medicine; DN, diabetic nephropathy; DMEM, Dulbecco’s modified Eagle’s medium; Cdc42, cell division cycle 42; HG, high glucose; BIS, basal insulin secretion; SPK, simultaneous pancreas/kidney.
