# Supplementary figures and images for: Cdc42 upregulation under high glucose induces podocyte apoptosis and impairs β-cell insulin secretion
Source: Front Endocrinol (Lausanne). 2022 Aug 10;13:905703. doi: 10.3389/fendo.2022.905703 (PMC9399854; doi:10.3389/fendo.2022.905703)

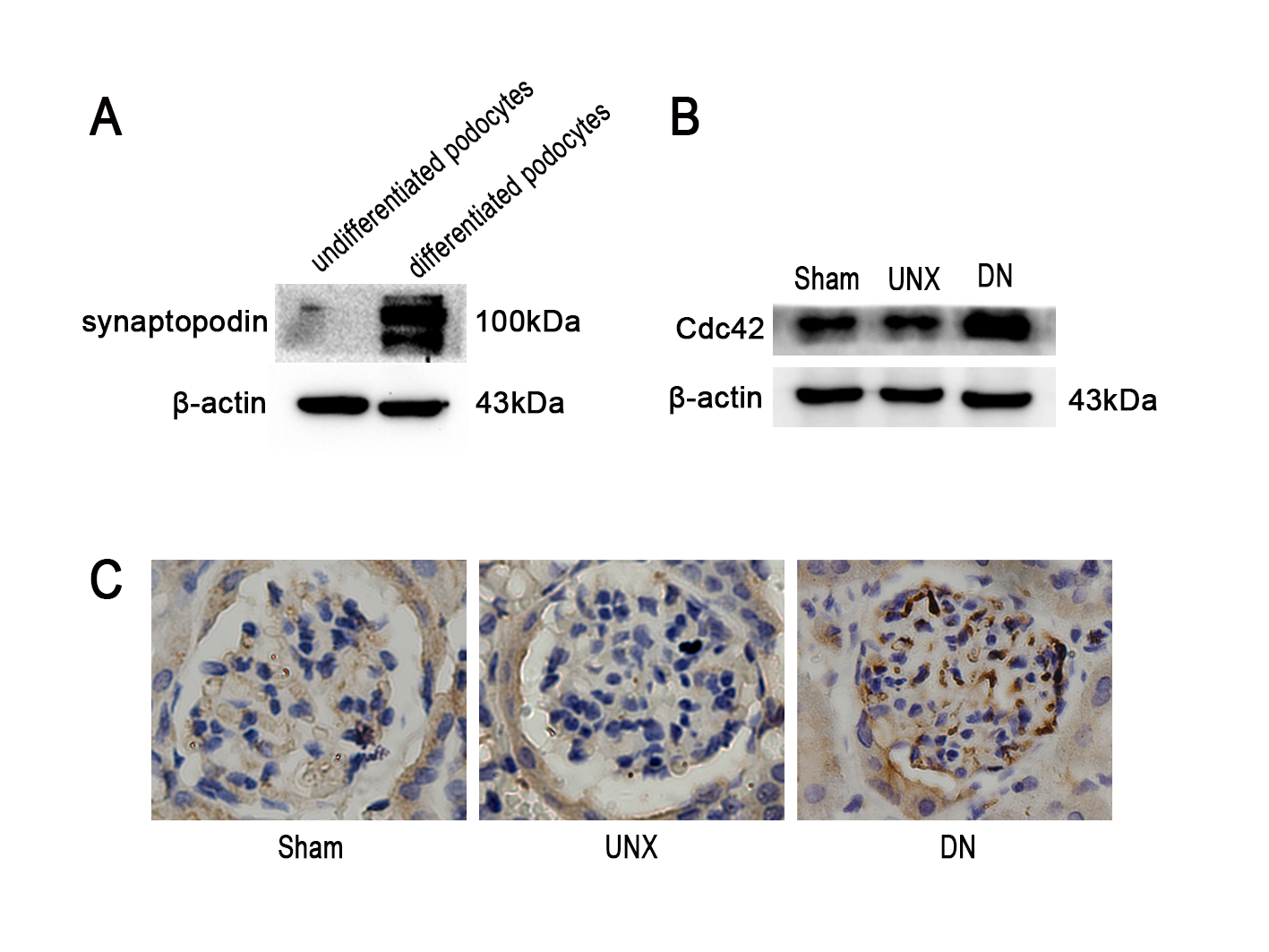

Supplement: Supplementary Figure 1 — Identification of MPC5 cell differentiation and the expression of Cdc42 in mice with or without diabetic nephropathy (DN). (A) The protein expression of synaptopodin. (B) The protein expression of Cdc42 in the UNX, Sham, and DN groups. (C) Immunohistochemical evaluation of Cdc42 in the UNX, Sham, and DN groups; original magnification: ×400. [file Image_1.tif]

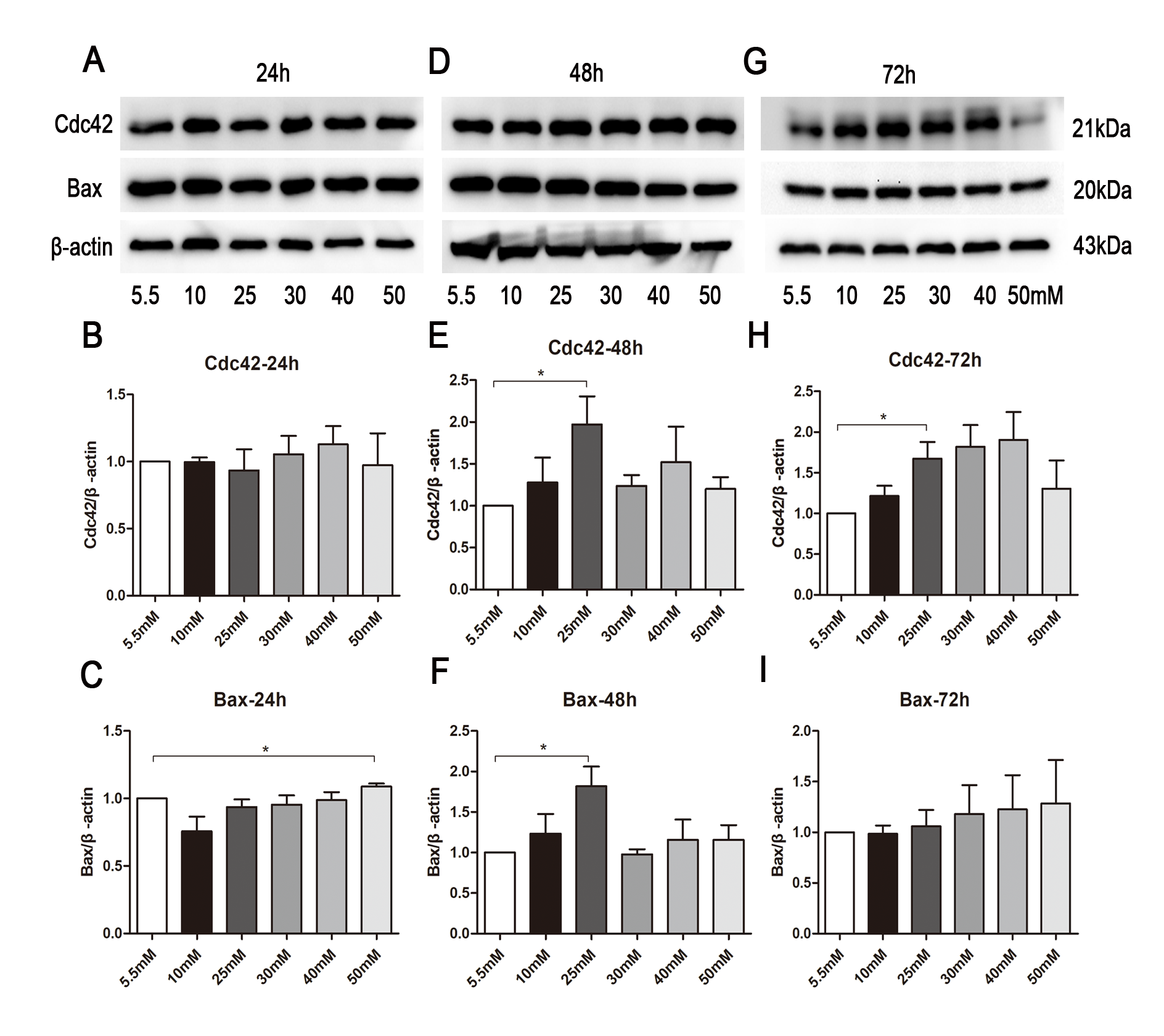

Supplement: Supplementary Figure 2 — The expression of Cdc42 and Bax at various glucose concentrations and timepoints. Cdc42 and Bax protein levels when treated with various glucose concentrations at different timepoints. High glucose (25 mM) significantly increased the abundance of Cdc42 and Bax compared with normal glucose. [file Image_2.tif]

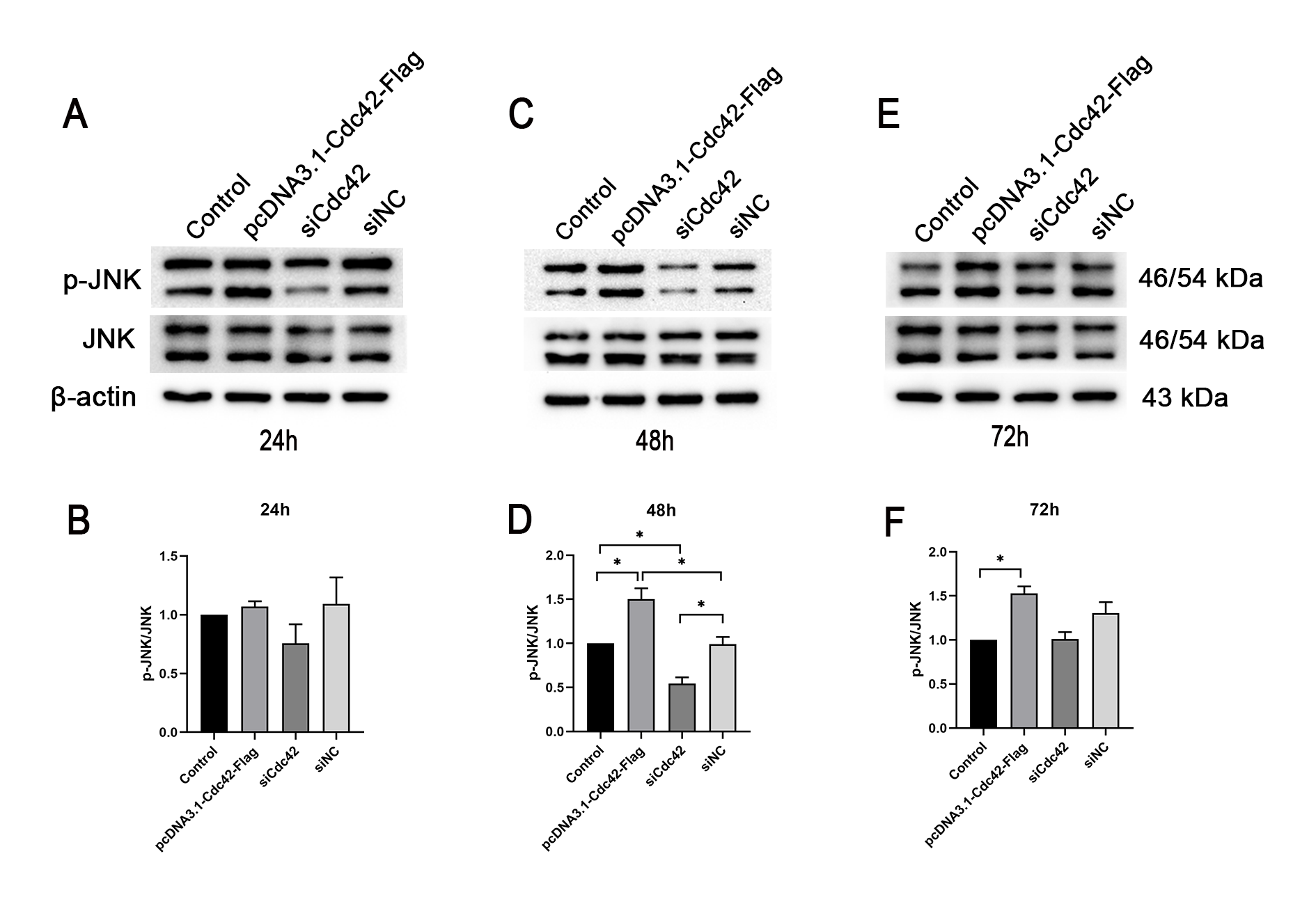

Supplement: Supplementary Figure 3 — Protein expression levels of JNK and p-JNK at different time gradients. The protein expression levels of JNK and p-JNK were detected at 24, 48, and 72 h after transfection. UNX, unilateral nephrectomy group; Sham, sham operation group; DN, diabetic nephropathy. The data are presented as mean ± SEM; *P < 0.05. [file Image_3.tif]
